# An Adequate Infusion Rate of Remimazolam for Induction of General Anesthesia in Adult Patients: A Prospective Up-and-Down Dose-Finding Study

**DOI:** 10.3390/jcm12051763

**Published:** 2023-02-22

**Authors:** Heejoon Jeong, Hara Kim, Hyun Joo Ahn

**Affiliations:** Department of Anesthesiology and Pain Medicine, Samsung Medical Center, Sungkyunkwan University School of Medicine, Seoul 06351, Republic of Korea

**Keywords:** remimazloam, induction, infusion rate, up-and-down method

## Abstract

Remimazolam is a recently developed anesthetic agent for general anesthesia and sedation. Currently, the optimal infusion rate for the induction of general anesthesia within two minutes remains unclear. We estimated the 50% and 90% effective doses (ED_50_ and ED_90_) of remimazolam required for loss of responsiveness within two minutes in adult patients using the up-and-down method. The starting infusion rate of remimazolam was 0.1 mg/kg/min and was increased or decreased by 0.02 mg/kg/min intervals in the following patient according to the success or failure of the previous patient. Success was defined as a loss of responsiveness within two minutes. Patient enrollment continued until six crossover pairs were observed. The ED_50_ and ED_90_ were estimated by centered isotonic regression and the pooled adjacent violators algorithm with bootstrapping, respectively. Twenty patients were included in the analysis. The ED_50_ and ED_90_ of remimazolam for loss of responsiveness within two minutes were 0.07 mg/kg/min (90% CI: 0.05, 0.09 mg/kg/min) and 0.10 mg/kg/min (90% CI: 0.10, 0.15 mg/kg/min), respectively. Vital signs were stable with an infusion rate of 0.10 mg/kg/min, and no patients required inotrope/vasopressor. Intravenous infusion of remimazolam at a rate of 0.10 mg/kg/min can be an effective approach to inducing general anesthesia in adult patients.

## 1. Introduction

An ideal induction agent is characterized by a fast onset, rapid recovery, and few adverse effects, such as circulatory depression [1,2]. Remimazolam is an ultra-short-acting intravenous benzodiazepine that was recently developed and approved for the induction and maintenance of general anesthesia or procedural sedation [3]. Remimazolam is an agonist at the gamma-aminobutyric acid receptor, which is rapidly hydrolyzed to a pharmacologically inactive metabolite by non-specific tissue esterase in the body [3,4]. In previous studies, it presented a high clearance rate, a small steady-state volume of distribution, and a short context-sensitive half-life of 7.5 min after 3 h of constant rate infusion [5,6,7]. In addition, remimazolam can be easily reversed by flumazenil and has no notable pain on injection [5,6,7,8].

Compared to propofol, remimazolam presented a more stable hemodynamic profile during and after the induction of anesthesia in previous studies. Patients who were induced by remimazolam showed a significantly lower incidence of hypotensive adverse events [9,10], and less need for vasopressors compared to patients who were induced by propofol [11]. Due to the advantages of hemodynamic stability, remimazolam is receiving significant attention as an induction agent for general anesthesia [9].

The manufacturer recommends intravenous administration of remimazolam at a rate of 0.1 mg/kg/min or 0.2 mg/kg/min, and adjustment of infusion rates according to the patient’s conditions for induction of general anesthesia. However, these infusion rates were determined to be doses that showed non-inferiority in terms of safety and efficacy compared to propofol [10], but not based on systematic dose-finding studies. Furthermore, these infusion rates are recommended when intravenous remifentanil is administered simultaneously. Because administering more anesthetics than necessary can cause hemodynamic instability during or after anesthetic induction, a precise dose guideline is important for patient safety. In addition, information on the appropriate induction dose as a sole hypnotic is required. Nevertheless, there has been no study on the optimal infusion rate of remimazolam as a sole hypnotic agent that is required to induce loss of responsiveness within 2 min, which is usually required for general anesthesia induction. Recently, Liu et al. [12] reported the median effective dose of remimazolam for induction of general anesthesia (0.061–0.088 mg/kg). However, they included only elderly patients aged over 60 years and used a bolus injection of remimazolam, which the manufacturer of remimazolam did not recommend because bolus dosing may cause large variability in remimazolam concentrations over time [6]. Moreover, this study was performed in patients who received remifentanil infusion simultaneously.

Since remimazolam has been approved only for continuous infusion, we focused on exploring the optimal infusion rate of remimazolam. We hypothesized that there would be a specific optimal infusion rate of remimazolam to induce loss of responsiveness within 2 min in adult patients while maintaining stable hemodynamics in inhalation anesthesia. We investigated the 50% and 90% effective doses of remimazolam using centered isotonic regression and the pooled adjacent violators algorithm (PAVA) with bootstrapping.

## 2. Materials and Methods

### 2.1. Study Design and Ethical Statement

This prospective dose-finding study was conducted at a tertiary hospital in South Korea between 14 January 2022 and 20 January 2022. Ethical approval for this study (SMC 2021-07-121-001) was provided by the Institutional Review Board of Samsung Medical Centre, Seoul, South Korea, on August 30, 2021. This study was registered at the Clinical Research Information Service before patient enrollment (identifier: KCT0006805; date of registration: 3 December 2021; principal investigator: Hyun Joo Ahn). Written informed consent was obtained from all participants. This study adhered to the ethical principles of the 1964 Declaration of Helsinki and its amendments. This manuscript adhered to the Transparent Reporting of Evaluations with Nonrandomized Designs (TREND) guidelines [13]. All methods were performed according to the approved guidelines.

### 2.2. Participants

Adult patients aged 20 to 65 years and with American Society of Anesthesiologists physical status I to II who were scheduled for elective surgery under general anesthesia with inhalation agents were assessed for eligibility. Exclusion criteria included major contraindications of remimazolam, as follows: known allergy or contraindication to benzodiazepine, severe liver disease, heavy alcohol consumption, neuromuscular disease, psychiatric disorders, metabolic disorders, acute narrow-angle glaucoma, and lactose intolerance. Patients who had severe sleep apnea disorder, who showed unstable vital signs before surgery, or who refused to participate were also excluded. According to the intention-to-treat principle, all participants assigned their infusion rate of remimazolam for the dose titration study were included in the analysis.

### 2.3. Study Protocol

The patients did not receive any premedication. After the patients were transferred to the operating room, standard monitors were applied to monitor vital signs, pulse oximetry, electrocardiography, and non-invasive blood pressure. In addition, a bispectral index (BIS) monitor (BIS^TM^, Medtronic, MN, USA) was used to assess the depth of anesthesia. Before induction of anesthesia, each patient performed spontaneous breathing with 100% oxygen for preoxygenation. After more than 2 min of preoxygenation with a fraction of inspired oxygen of 1.0, an intravenous infusion of remimazolam was started at a rate that was allocated to each patient according to the study protocol.

Remimazolam was prepared at a concentration of 0.1 mg/mL, and its predetermined dose was administered as an infusion using a syringe pump (Agilia MC, Fresenius Kabi AG, Bad Homburg, Germany). The infusion rate in each patient was determined based on Dixon and Mood’s up-and-down sequential method [14]. The first patient received remimazolam at a rate of 0.1 mg/kg/min, the lowest infusion rate recommended by the manufacturer for induction of general anesthesia. The infusion rate for the next patient was determined based on whether the induction of anesthesia in the previous patient succeeded or failed. If the previous patient showed success, the subsequent patient received a infusion reduced by 0.02 mg/kg/min. Conversely, if the previous patient showed a failure, the following patient received an infusion increased by 0.02 mg/kg/min. Because Dixon’s sequential method requires six or more crossover pairs for statistical analysis, the recruitment of patients continued until six failure–success crossover pairs were observed.

A designated study staff member (anesthesiologist), who was blinded to the dose of remimazolam, checked the patient for loss of responsiveness for 2 min from the start of remimazolam infusion. No other anesthetic agents, opioids, or neuromuscular blocking agents were administered during this period. However, if the patient’s blood pressure decreased by more than 20% from the baseline during remimazolam infusion, 5 mg of ephedrine was administered. If a heart rate of < 45 beats/min was observed, atropine (0.5 mg) was administered. Time to loss of consciousness was recorded for 2 min from the start of remimazolam infusion. The blood pressure, heart rates, oxygen saturation, and BIS of all patients were recorded prior to remimazolam administration, at the time of loss of responsiveness, and every minute until 5 min after intubation. The number of inotropes/vasopressors given during remimazolam infusion was recorded.

Loss of responsiveness was assessed from the start of remimazolam infusion as follows: The patient was asked to count from 1 to 100 from the start of remimazolam infusion. When the patient stopped counting, the study staff slightly shook the patient’s shoulders and asked them to open their eyes. Loss of responsiveness was defined as no response to gentle touch and verbal command. Success was defined as loss of responsiveness within 2 min from the beginning of remimazolam infusion based on the manufacturer’s recommendations. It was considered a failure if the patient did not lose responsiveness for 2 min. In failure cases, 1 mg/kg of intravenous propofol was administered to induce a loss of responsiveness. Failure cases were included for the determination of the 50% and 90% effective doses (centered isotonic regression and PAVA with bootstrapping) and analysis of hemodynamic variables but excluded from calculating the time required for loss of responsiveness.

### 2.4. Anesthesia Protocol

After the patients were confirmed as having loss of responsiveness (when the dose titration study ended), remimazolam infusion was discontinued until intubation while sevoflurane was administered. The attending anesthesiologist fitted the bag valve mask on the patients and performed manual ventilation with 5 vol% of sevoflurane in a 3:1 mixture of oxygen and air. After confirming proper ventilation without any difficulties, intravenous rocuronium (0.6 mg/kg) and fentanyl (25 µg) were administered to all patients. After more than 2 min, endotracheal intubation was performed carefully. Volatile anesthesia using sevoflurane was maintained to achieve a BIS value of 40–60. During the induction and maintenance of anesthesia, if the blood pressure decreased by 20% from the baseline, intravenous ephedrine (5 mg) was administered. If the heart rate decreased to less than 45 beats/min, intravenous atropine (0.5 mg) was administered.

For postoperative analgesia, intravenous hydromorphone (0.01 mg/kg) and paracetamol (1 g) were administered 10 min before the end of surgery. Neuromuscular blockade was reversed with intravenous sugammadex (2–4 mg/kg) based on the number of train of four. Extubation was performed when the patient recovered adequate muscle strength, and all patients were transferred to the post-anesthesia care unit. Before discharge from the post-anesthesia care unit, the study nurse who was blinded to the allocated infusion rate and success/failure of the study assessed intraoperative awareness using a modified Brice questionnaire (Table 1).

### 2.5. Blinding Method

Remimazolam was prepared at the same concentration in all cases. Before the patient and anesthesiologist arrived in the operating room, the corresponding author set the infusion pump for each patient according to the infusion rate determined by the up-and-down method. Additionally, the infusion pump was then covered to maintain blindness. Therefore, the infusion rate of remimazolam in each patient was blinded to the patient, the designated study staff who assessed and determined the loss of responsiveness, and the study nurse who evaluated intraoperative awareness in the post-anesthesia care unit. After induction of anesthesia, the covered infusion pump was retrieved and the total amount of remimazolam administration was identified by the corresponding author.

### 2.6. Outcome Measurements

The primary endpoint was the 50% effective dose and 90% effective dose of remimazolam infusion rate, which was required for loss of responsiveness within 2 min from the start of infusion in adult patients. The secondary endpoints included mean blood pressure, heart rate, and the BIS value at various time points (before induction of anesthesia, at the time of loss of responsiveness, and every minute until 5 min after intubation). Other secondary outcomes included the time interval between the start of remimazolam infusion and the loss of responsiveness, the total infused volume of remimazolam in success cases, the number of patients that experienced hypotension (mean blood pressure) < 65 mmHg, hypertension (mean blood pressure > 140 mmHg), bradycardia (heart rate < 45 beats/min), intraoperative awareness, which was investigated using a modified Brice questionnaire, and the number of patients who required an inotrope or vasopressor during the induction of anesthesia.

### 2.7. Statistical Analysis

The 50% effective dose and corresponding 90% confidence interval (CI) of remimazolam for inducing loss of responsiveness within 2 min were calculated using Dixon and Mood’s up-and-down method with a centered isotonic regression [14,15]. A priori calculation of the sample size was not conducted. For statistical analysis using Dixon and Mood’s up-and-down method, at least six failure–success crossover pairs were required. Thus, patient enrollment continued until six crossover pairs were observed. Because Dixon and Mood’s up-and-down method is also known to need 40 or more cases, we planned to recruit 44 patients considering the 10% dropout rate, but the consecutive enrollment can be stopped early if six crossover pairs are observed.

The 90% effective dose and corresponding 90% CI were calculated using a centered PAVA with bootstrapping [15,16]. Unless the sample size is 100 or more, the up-and-down design generally estimates a 90% CI instead of a 95% CI [17]. Moreover, dose estimation using the centered isotonic regression and centered PAVA method guarantees sufficient coverage only up to 90% effective dose at a typical up-and-down design and sample size [17]. Therefore, we investigated a 90% effective dose and corresponding 90% CI in this study.

Continuous variables are summarized as medians (interquartile range). Categorical variables are summarized as numbers (%). The secondary outcomes were evaluated using a Mann–Whitney U test for continuous variables. Repeated measures analysis of variance (ANOVA) was performed to compare the change in hemodynamic variables and the BIS value between the groups. Two-sided *p*-values < 0.05 were considered statistically significant. Statistical analyses were performed using SPSS version 22 (SPSS Inc., Chicago, IL, USA) and R statistical software version 3.3.2 (Vienna, Austria; http://www.R-project.org.

## 3. Results

Twenty-three patients were assessed for eligibility, but three patients declined to participate. When twenty consecutive patients were enrolled in this trial, six crossover pairs were observed. Therefore, enrollment was stopped according to the study protocol and all twenty patients were included in the estimation of 50% and 90% effective doses that induced loss of responsiveness within 2 min. No patients were excluded from the study. The demographic characteristics of the participants are presented in Table 2.

**Figure 1:**
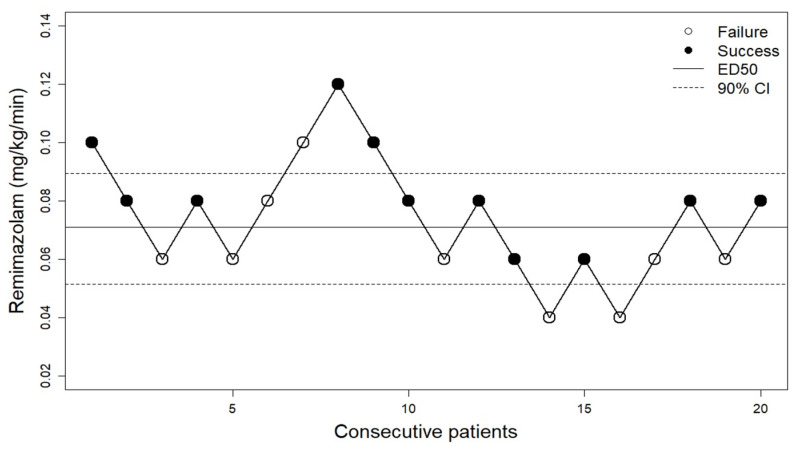
The up-and-down sequence of remimazolam dose for inducing loss of responsiveness within two minutes from the start of infusion. The patient sequence number (*x*-axis) is the ordering of patient exposures. Success: loss of responsiveness within 2 min from the start of remimazolam infusion. Failure: no loss of responsiveness within 2 min from the start of remimazolam infusion. ED50, 50% effective dose; CI, confidence interval.

**Figure 2:**
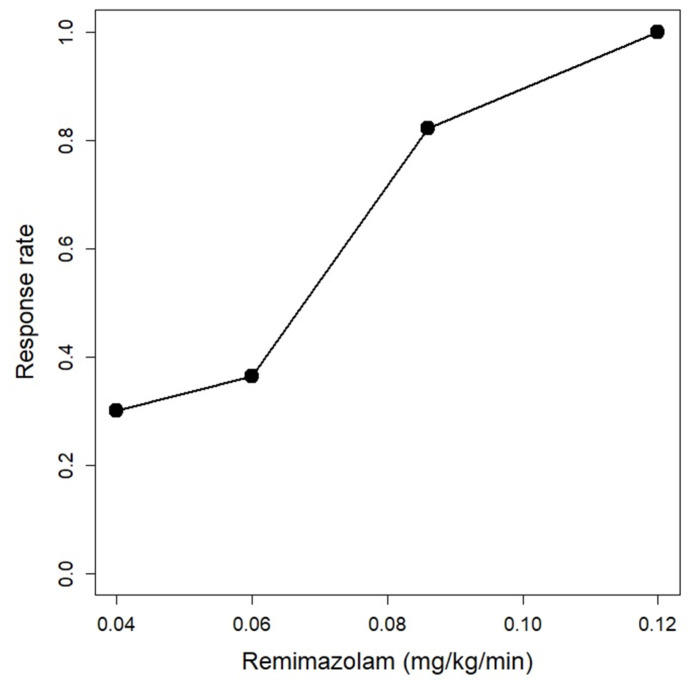
Centered pooled adjacent violators algorithm (PAVA) response rate. It predicted that an infusion rate of 0.10 mg/kg/min could induce loss of responsiveness within two minutes from the start of infusion in 90% of patients.

Assessment of success or failure of loss of responsiveness for consecutive patients is shown in the up-and-down sequence of Figure 1. The 50% effective dose of remimazolam for loss of responsiveness was estimated using centered isotonic regression to be 0.07 mg/kg/min (90% CI: 0.05, 0.09), and the 90% effective dose was estimated using the centered PAVA method to be 0.10 mg/kg/min (90% CI: 0.10, 0.15) (Figure 2).

A total of 11/20 patients successfully showed a loss of responsiveness via remimazolam infusion at rates assigned to each of them. Among the patients who successfully lost responsiveness, the median [interquartile range] total infused dose of remimazolam need to reach loss of responsiveness was 13 [10,11,12,13] mg, which is equivalent to 0.2 [0.2–0.2] mg/kg of ideal body weight. The median duration from the start of the infusion to the loss of responsiveness was 95 [77–100] seconds (Table 3).

Secondary outcomes including hemodynamic change, BIS value, and other study data between the success group (*n* = 11) and failure group (*n* = 9) are also presented in Table 3. Despite the various infusion rates for each patient, none of the patients experienced hypotension or bradycardia that required rescue medication, and the median blood pressure was maintained within 20% of the baseline in all patients successfully induced by remimazolam (Table 3). No adverse events such as injection pain were reported during the study period.

No intraoperative awareness was observed during the study. None recalled events after induction or dreaming during surgery. No adverse events related to the study protocols were observed.

## 4. Discussion

Using the up-and-down design, this study showed that the 50% and 90% effective remimazolam infusion rates to induce loss of responsiveness within 2 min in adult patients were 0.07 mg/kg/min (90% CI: 0.05, 0.09) and 0.10 mg/kg/min (90% CI: 0.10, 0.15), respectively. During remimazolam induction, blood pressure and heart rates were maintained within 20% of the baseline values and no patients required vasoactive medications.

Remimazolam is a recently approved ultra-short-acting intravenous benzodiazepine for general anesthesia or procedural sedation [3]. Despite the growing interest in remimazolam, with its recent approval, few studies have characterized the 50% and the 90% effective remimazolam infusion rates for induction of general anesthesia. The manufacturer recommends infusion rates of 0.1 mg/kg/min and 0.2 mg/kg/min for the induction of general anesthesia based on phase II/III clinical trials. However, these phase II/III clinical trials were intended to demonstrate the safety and efficacy of remimazolam for general anesthesia and sedation compared to propofol and were not dose titration studies [10]. Nevertheless, most previous studies chose either 0.1 mg/kg/min or 0.2 mg/kg/min to induce loss of responsiveness based on the manufacturer’s recommendation [18,19]. We tried to explore a more systematic method to find adequate infusion rates of remimazolam for induction of general anesthesia in adult patients. In general, the estimation of the dose for new agents is aimed at the minimum dose, which provides a 50% probability of response, i.e., a 50% effective dose. Thus, the Dixon and Mood up-and-down method that targets a 50% effective dose is commonly used for dose-finding studies of fast-response drugs such as anesthetics [16,20]. However, identifying a 90% or higher effective dose is clinically more important, as 50% efficacy is an inappropriately low threshold for anesthesia. To determine the 90% effective dose and corresponding 90% CI, we used a centered PAVA and bootstrapping [15,16].

Most previous studies administered remimazolam in combination with remifentanil for the induction of anesthesia. When remimazolam is combined with remifentanil, the recommended infusion rates for anesthesia induction may not be the same as the recommended infusion rate of remimazolam alone. In this study, we estimated the 90% effective infusion rate to be 0.1 mg/kg/min. This result means that the previously recommended infusion rate of remimazolam is relatively high, or that the effect of remifentanil on the loss of responsiveness is not significant when remimazolam is used for induction of anesthesia.

This study also evaluated the hemodynamic stability after remimazolam induction. Hypotension frequently occurs during and after anesthetic induction and can threaten patient safety [21]. For this reason, anesthesiologists try to minimize hemodynamic changes during and after the induction of anesthesia. Remimazolam is known to preserve hemodynamics more stably than propofol. A randomized controlled trial reported that the patients who received remimazolam infusion at both the rates of 0.1 and 0.2 mg/kg/min showed a lower incidence of hypotension compared with the patients who received an injection of 2 mg/kg of propofol bolus [10]. The incidence of adverse events, such as hypotension, bradycardia, and low oxygen saturation, was significantly lower in the continuous remimazolam infusion group than in the propofol group [8,22,23]. In addition, Hasegawa et al. [24] reported that remimazolam showed a lower variability of heart rate during the induction of anesthesia, preserving the balance of sympathetic and parasympathetic activities, while propofol modulated it to sympathetic dominance. In our study, patients induced with 0.10 mg/kg/min of remimazolam (90% effective dose as estimated in this study) did not show severe hypotension, tachycardia, or bradycardia compared to the baseline.

Generally, a bolus injection of hypnotics is more practical and preferred during the induction of anesthesia. Indeed, although bolus administration of remimazolam has not been approved, several published trials have explored bolus dosing of remimazolam for induction of general anesthesia. In these trials, the average time from the start of remimazolam injection to loss of responsiveness was 100 s [10,25], with doses of 0.2–0.4 mg/kg [9,26]. According to our study, the median [interquartile range] total infused amount of remimazolam was 0.2 [0.2–0.2] mg/kg in successful cases, and the median time to loss of responsiveness was 95 [77–100] seconds. Although remimazolam boasts stable hemodynamics, previous studies have shown that bolus injection of remimazolam can result in dose-dependent hypotension. In fact, a remimazolam bolus of 0.4 mg/kg showed a similar incidence of hypotension when compared to a propofol bolus of 2 mg/kg [9]. Our study suggests that 0.2 mg/kg may be used as an adequate bolus dose among the proposed doses in previous studies if a continuous infuser is not available.

In this study, we defined the loss of responsiveness as no response to mild shaking of the patient’s shoulder. This definition corresponds to the Modified Observer’s Assessment of Alertness/Sedation (MOAA/S) scale score 1. The MOAA/S scale is frequently used to describe the depth of sedation and anesthesia [27]. However, an adequate MOAA/S scale for anesthesia induction is controversial. Previous studies adopted the MOAA/S scale score 0 (no response after a painful trapezius squeeze) as an appropriate state of loss of consciousness [8,9,12] or the MOAA/S scale score 1 like ours [10,18]. The MOAA/S scale score 1 may not be sufficient for invasive procedures such as endotracheal intubation. However, opioids and other hypnotics (inhalation agents) usually follow shortly after the loss of consciousness in clinical anesthesia practice. In addition, a higher amount of hypnotics, which produces more rapid and deeper sedation than necessary, is likely to cause hemodynamic instability during the induction of anesthesia.

The present study has some limitations. First, this study included relatively healthy patients with an American Society of Anesthesiologists physical status of I and II. The lack of diversity in the population can limit the generalizability of our findings. The response to remimazolam can vary depending on the patient’s underlying condition. Therefore, further studies are warranted in elderly patients or in patients with compromised circulatory function with an American Society of Anesthesiologists physical status III or higher to confirm the safety and efficacy of remimazolam in such patients. Second, we used a classical up-and-down design with centered isotonic regression and PAVA with bootstrapping with a relatively small sample size. Although it is reasonable to estimate a 50% effective dose and our results are consistent with those of previous studies, a fixed-staircase or biased-coin up-and-down design including more samples could be appropriate to estimate a highly effective dose, such as a 95% effective dose [17]. Third, the study was designed to evaluate the success or failure of loss of consciousness within 2 min after remimazolam infusion as a sole anesthetic agent. Therefore, the appropriate dose or hemodynamic stability of remimazolam when administered in combination with other anesthetic agents, such as opioids and neuromuscular blockers, was not evaluated. Fourth, remimazolam was administered during the induction period only. Since general anesthesia was maintained using sevoflurane, the result of the intraoperative awareness assessment was also influenced by the inhalation agent. Fifth, the BIS value was not used to determine the loss of responsiveness in this study due to the paucity of evidence that the BIS value had a significant correlation with the level of consciousness during the induction with intravenous remimazolam. However, further studies are required to show that BIS decreases as the remimazolam infusion rate increases and that sedation due to remimazolam is closely correlated to the BIS values. Sixth, infusion rates may be different when a target-controlled infusion (TCI) pump is used. Further studies are required using different types of infusion.

## 5. Conclusions

In conclusion, continuous infusion of remimazolam at a rate of 0.10 mg/kg/min can be an effective approach to induce general anesthesia in adult patients within 2 min (median of 95 s) without untoward hemodynamic side effects.

## Figures and Tables

**Table 1:** A modified Brice questionnaire.

1. What is the last thing you remember before surgery? 2. What is the first thing you remember after waking up? 3. Can you recall anything between being under anesthesia and waking up? 4. Did you dream of anything during surgery? If so, was it disturbing? 5. What did you find most unpleasant about the surgery?

**Table 2:** Baseline characteristics of participants.

Variables	*n* = 20
Age, years	50 [46–54]
Sex, male/female	8/12
Body mass index, kg/m^2^	22.0 [20.4–26.6]
American Society of Anesthesiologists physical status (I/II)	14/6
Comorbidity	
Hypertension	2 (10)
Diabetes mellitus	4 (20)
Duration of surgery, min	112 [96–151]
Type of surgery	
Abdominal surgery	7 (35)
Breast surgery	10 (50)
Thyroidectomy	3 (15)

Data are presented as median [interquartile range] or number (%).

**Table 3:** Hemodynamic changes and secondary outcomes between the groups.

Variables	Success Group (*n* = 11)	Failure Group (*n* = 9)	*p*-Value ^a^
Time interval from the start of infusion to loss of responsiveness, sec	95 [77–100]	NA	
Total amount of remimazolam, mg	13 [10–13]	NA	
Patients who needed vasoactive medication during induction	0	0	
During the induction of anesthesia			
Mean blood pressure, mmHg			0.533
Before induction of anesthesia	97 [85–107]	91 [86–104]	
At the loss of responsiveness	87 [80–100]	77 [68–99]	
After 1 min after intubation	117 [93–129]	96 [78–124]	
After 3 min after intubation	105 [90–112]	87 [83–124]	
After 5 min after intubation	98 [88–106]	94 [85–100]	
Heart rate, beats/min			0.581
Before induction of anesthesia	74 [66–78]	76 [68–87]	
At the loss of responsiveness	76 [63–95]	80 [77–91]	
After 1 min after intubation	94 [82–96]	104 [92–108]	
After 3 min after intubation	90 [86–101]	94 [93–111]	
After 5 min after intubation	96 [85–105]	82 [84–100]	
Bispectral index			0.428
Before induction of anesthesia	93 [89–97]	94 [91–97]	
At the loss of responsiveness	56 [48–69]	57 [41–66]	
After 1 min after intubation	57 [40–61]	45 [30–59]	
After 3 min after intubation	48 [36–59]	39 [29–59]	
After 5 min after intubation	40 [39–53]	50 [28–55]	
Patients who experienced intraoperative awareness	0	0	

Data presented as median [interquartile range] or number (%). ^a^ *p*-value was calculated using the repeated measures analysis of variance (ANOVA).

## Data Availability

The data presented in this study are available on request from the corresponding author.
